# Analysis of the Role of Interleukin 6 Receptor Haplotypes in the Regulation of Circulating Levels of Inflammatory Biomarkers and Risk of Coronary Heart Disease

**DOI:** 10.1371/journal.pone.0119980

**Published:** 2015-03-17

**Authors:** Bruna Gigante, Rona J. Strawbridge, Ilais Moreno Velasquez, Zahra Golabkesh, Angela Silveira, Anuj Goel, Damiano Baldassarre, Fabrizio Veglia, Elena Tremoli, Robert Clarke, Hugh Watkins, Anders Hamsten, Steve E. Humphries, Ulf de Faire

**Affiliations:** 1 Unit of Cardiovascular Epidemiology, Institute of Environmental Medicine (IMM), Stockholm, Sweden; 2 Division of Cardiovascular Medicine, Department of Clinical Sciences, Danderyd University Hospital, Stockholm, Sweden; 3 Cardiovascular Genetics and Genomics Group, Atherosclerosis Research Unit, Department of Medicine Solna, all at Karolinska Institutet, Stockholm, Sweden; 4 Department of Cardiovascular Medicine, The Wellcome Trust Centre of Human Genetics, University of Oxford, Oxford, United Kingdom; 5 Dipartimento di Scienze Farmacologiche e Biomolecolari, Università di Milano, Milan, Italy; 6 Centro Cardiologico Monzino, IRCCS, Milan Italy; 7 Clinical Trial Service Unit, University of Oxford, Oxford, United Kingdom; 8 Dept of Cardiology, Karolinska University Hospital, Stockholm, Sweden; 9 Centre for Cardiovascular Genetics, University College London, United Kingdom; University of Florida, UNITED STATES

## Abstract

Variants at the interleukin 6 receptor (*IL6R*) gene regulate inflammation and are associated with risk of coronary heart disease (CHD). The aim of the present study was to investigate the effects of *IL6R* haplotypes on circulating levels of inflammatory biomarkers and risk of CHD. We performed a discovery analysis in SHEEP, a myocardial infarction (MI) case control study (n = 2,774) and replicated our results in two large, independent European populations, PROCARDIS, a CHD case control study (n = 7,998), and IMPROVE (n = 3,711) a prospective cardiovascular cohort study. Two major haplotype blocks (rs12083537A/G and rs4075015A/T—block 1; and rs8192282G/A, rs4553185T/C, rs8192284A/C, rs4240872T/C and rs7514452T/C—block 2) were identified in the *IL6R* gene. *IL6R* haplotype associations with C-reactive protein (CRP), fibrinogen, IL6, soluble IL6R (sIL6R), IL6, IL8 and TNF-α in SHEEP, CRP and fibrinogen in PROCARDIS and CRP in IMPROVE as well as association with risk of MI and CHD, were analyzed by THESIAS. Haplotypes in block 1 were associated neither with circulating inflammatory biomarkers nor with the MI/CHD risk. Haplotypes in block 2 were associated with circulating levels of CRP, in all three study populations, with fibrinogen in SHEEP and PROCARDIS, with IL8 and sIL6Rin SHEEP and with a modest, non significant, increase (7%) in MI/CHD risk in the three populations studied. Our results indicate that *IL6R* haplotypes regulate the circulating levels of inflammatory biomarkers. Lack of association with the risk of CHD may be explained by the combined effect of SNPs with opposite effect on the CHD risk, the sample size as well as by structural changes affecting sIL6R stability in the circulation.

## Introduction

The interleukin 6 receptor gene *(IL6R)* region centered at rs8192284A/C, a functional SNP causing a Asp358Ala aminoacid change in the membrane bound IL6R, has been associated with the risk of coronary heart disease (CHD) [1–4], as well as with serum levels of inflammatory biomarkers. In particular, the C allele is associated with lower levels of C-reactive protein (CRP), fibrinogen [5] and with higher levels of interleukin 6 (IL6) [6] and soluble interleukin 6 receptor (sIL6R) [3,4]. These data indicate that *IL6R* may regulate inflammation and atherosclerosis. However the underlying mechanisms are still largely unknown.

Association of single SNPs with complex diseases leaves unexplored the effect exerted by other genetic variants mapping on the same gene. In this respect haplotypes may be considered as “super-alleles”, where the different genetic variants act in synergy [7,8]. Since the effect size of each genetic variant on complex phenotypes is rather small, haplotypes may provide further insights into mechanisms underlying the association of candidate genes with complex and intermediate phenotypes [9].

To disentangle the association of *IL6R* with inflammation and risk of CHD, we analyzed the association of *IL6R* haplotypes with circulating levels of inflammatory biomarkers [CRP, fibrinogen, sIL6R, IL6, interleukin 8 (IL8) and TNF-α] and with the risk of myocardial infarction (MI) and CHD. We performed our discovery study in the Stockholm Heart Epidemiology study (SHEEP), a large case control study conducted in the greater Stockholm area, and replicated the main findings in two independent, multicenter European studies: the Precocious Coronary Artery Disease (PROCARDIS) study [10] and the carotid Intima-Media thickness (c-IMT) and c-IMT Progression as Predictors of Vascular Events in a high risk European population (IMPROVE) study [11].

## Materials and Methods

### Study Populations

The SHEEP study is a population based case-control study designed to dissect both genetic and environmental factors associated with the risk of MI [12]. Study design and population characteristics have been fully reported elsewhere [13]. In the present investigation, only cases diagnosed with a MI, who survived at least 28 days (n = 1213) and age- and sex-matched controls (n = 1561) were included. Measurements of hs-CRP, and TNF-α in serum and fibrinogen in plasma were performed as described [14]. IL6, sIL6R and IL8 levels were measured in serum using assays provided by MesoScale Diagnostic (Human Cytokine Assay, Ultra-Sensitive Kit, MSD, Bethesda, USA), following the manufacturer´s protocol.

The PROCARDIS study recruited CHD cases and controls from Sweden, Germany, the United Kingdom and Italy [10]. Briefly, cases (n = 5689) were defined as having had a diagnosis of MI or acute coronary syndrome before the age of 66 years. Control subjects (n = 2308) were unrelated individuals, free from symptoms of CHD up to the age of 66 years and without siblings diagnosed with CHD before 66 years. CRP and fibrinogen were measured as described [10].

IMPROVE is a multicenter longitudinal European study, designed to investigate whether progression of carotid IMT is an independent predictor of cardiovascular events. A detailed description of the study has been reported elsewhere [11]. Briefly, 3711 subjects, who were asymptomatic for cardiovascular disease but had at least three established cardiovascular risk factors (dyslipidemia, hypertension, diabetes, smoking and family history of cardiovascular disease), were recruited in five European countries (Finland, Sweden, the Netherlands, France, Italy). Incident cardiovascular events (n = 213) were recorded during a 3-year follow-up period. Genotype data were available for 3516 individuals (cases: 198 referent group: 3318). Hs-CRP was measured as described [11].

### Ethics

The SHEEP study was carried out in accordance with the Helsinki Declaration and approved in 1991 by the Regional Ethics Review Board at Karolinska Institutet, Stockholm, Sweden. All the study participants in the SHEEP gave their informed oral consent to be enrolled in the study, since at the time the study was initiated (1992) no forms for the written consent were available or in current use. Cases were informed about the study at the time they were discharged from the hospital and controls were informed via mail. Participation was voluntary and involved no evident risks and only study participants who agreed to participate in the study received the questionnaire and were invited for the medical examination. This consent procedure was approved by the Regional Ethics Review Board at Karolinska Institutet.

The PROCARDIS study was funded by the European Commission Framework 6 (FP6) program. The study was carried out in accordance with the Helsinki Declaration and approved by the Institutional Review Board (IRB) at each one of the 4 recruiting centers: (1) the Regional Ethics Review Board at Karolinska Institutet, Stockholm in Sweden, (2) the IRB at the University of Munster, Munster, in Germany, (3) the IRB at the Mario Negri Institute, Milano in Italy and (4) the IRB at the University of Oxford, Oxford, United Kingdom. All study participants provided their written informed consent to participate in the study, a procedure approved by each one of the local ethical committee. A complete list of the participating and contributing centers is available at http://www.procardis.org.

The IMPROVE study was funded by the V^th^ European Union (EU) program. The study was carried out in accordance with the Helsinki Declaration and approved by the IRB at each one of the seven recruiting centers: (1) the Regional Ethics Review Board at Karolinska Institutet, Stockholm Sweden, (2) IRB at the Groupe Hôpitalier Pitie-Salpetriere, Paris, France, (3) the IRB Comitato Etico delle Aziende Sanitarie della regione Umbria, Perugia and (4) the IRB at the Ospedale Niguarda Ca´Granda, Milano, both in Italy, (5) the IRB at the University Hospital Groningen, Groningen, the Netherlands, (6) the IRB Hospital District of Northern Savo and (7) and the IRB at University of Eastern Finland, both in Kuopio, Finland. Each participant provided two different written consents one for general participation in the study and one for genotyping.

### Single Nucleotide Polymorphism (SNP) genotyping and haplotype generation

#### SNP selection, genotyping and definition of haplotype blocks in the discovery cohort, the SHEEP study

Ten SNPs were originally genotyped by MALDI-TOF (Matrix Adsorbed Laser Desorption-Ionisation-Time Of Flight) [15], on the Massarray Analyzer platform (Sequenom, Mutation Analysis Facility at Karolinska Institutet) based upon HapMap data release 24 Nov08 (rs4845617A/G at the 5´UTR, rs12083537A/G, rs12090237G/A, rs4075015A/T, rs4601580A/T, rs8192282G/A, rs4553185T/C, rs4537545C/T, rs4240872T/C and rs7514452T/C at the 3´UTR). Genotype data were integrated with eight additional SNPs genotyped in the SHEEP included in the Illumina 200K CardioMetabochip [16] and mapping at *IL6R* (rs7553796C/A, rs7518199C/A, rs4453032A/G, rs4845625C/T, rs6689393G/A, rs4129267C/T, rs8192284A/C and rs4072391C/T).

**Fig 1:**
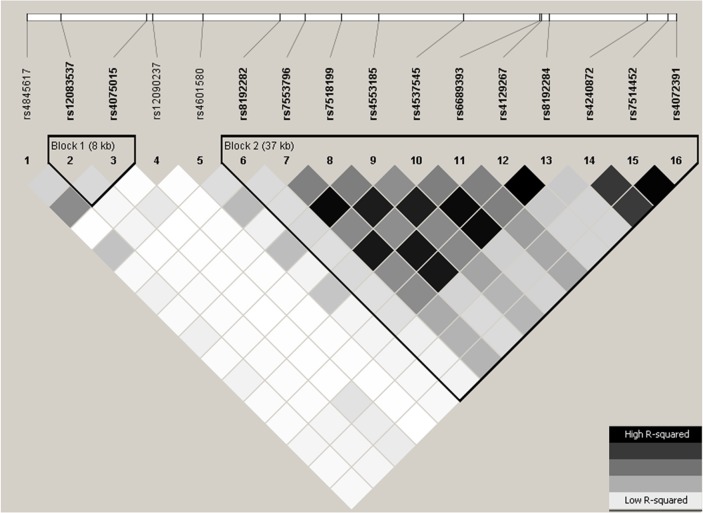
Representative LD plot of the two haplotype blocks at *IL6R* in SHEEP. Two of the 18 SNPs are not shown: rs4537545 in full LD with rs8192284 and rs4845625 in full LD with rs6689393 (both r2>0.95).

*IL6R* haplotype blocks were generated by Haploview [17], using the algorithm proposed by Gabriel [18]. Two haplotype blocks were identified in the SHEEP study (Fig. 1). The Haploview tagger function was the used to identify redundant SNPs. SNPs were considered redundant if the pairwise LD (r2) was ≥0.8.

Rs12083537A/G and rs4075015A/T were retained in the first haplotype block (block 1) and rs8192282G/A, rs4553185T/C, rs8192284A/C, rs4240872T/C and rs7514452T/C in the second haplotype block (block 2). Three SNPs (rs4845617A/G, rs12090237G/A and rs4601580A/T) did not belong to any of the two blocks.

#### Replication in the PROCARDIS and IMPROVE study

To replicate this analysis, fourteen *IL6R* SNPs genotyped with the Illumina 1M and 610K chips and available in the PROCARDIS study (rs12083537A/G, rs4075015A/T, rs12090237G/A, rs4601580A/T, rs8192282G/A, rs7553796C/A, rs7518199C/A, rs4553185T/C, rs4537545C/T, rs4129267C/T, rs8192284A/C, rs4240872T/C, rs7514452T/C and rs4072391C/T) were also run in Haploview. The two haplotype blocks were confirmed in the PROCARDIS study. The tagger function retained rs12083537A/G and rs4075015A/T in the haplotype block 1 and rs8192282G/A, rs4553185T/C, rs8192284A/C, rs4240872T/C in haplotype block2. To be consistent with the analysis performed in the SHEEP, the SNP rs7514452T/C was also retained even if redundant.

Finally, eight *IL6R* SNPs genotyped with the Illumina 200K CardioMetabochip (rs7553796C/A, rs7518199C/A, rs4453032A/G, rs4845625C/T, rs6689393G/A, rs4129267A/C, rs8192284A/C and rs4072391C/T) mapping in the haplotype block 2 generated in the SHEEP and PROCARDIS were available for the IMPROVE. The Haploview tagger function identified rs7553796 C/A (pairwise LD with rs4553185T/C, r2 = 0.96), rs8192284A/C and rs4072391T/C (pairwise LD with rs7514452T/C r2 = 0.98) as tagSNPs in the IMPROVE.

#### Haplotype generation in the SHEEP and in the replication cohorts

Haplotypes in block 1 and block 2 were inferred from the original genotype data using THESIAS v 3.1 [19] in the SHEEP and in the two replication cohorts. To control the correct inference of the haplotype from the genotype data, the haplotypic r2 value command in THESIAS was used and a r2 = 0.7 was set as cut off value, according to the program instructions. Haplotypes with a frequency ≤1% were excluded from the analysis. The haplotypic r2 value and the haplotype frequencies in the three study populations are reported in S1 Table.

### Statistical Analysis

Continuous variable are reported as mean and 95% confidence interval (CI), binary variables are reported as percentages.

Haplotypes were inferred from the original genotype data and analyzed for association with the circulating levels of inflammatory biomarkers and for association with the MI/CHD risk using THESIAS v 3.1 [19].

Haplotypes were tested for association with serum/plasma levels of inflammatory biomarkers [CRP (mg/L), fibrinogen (g/L), IL6 (ng/ml), sIL6R (ng/ml), IL8 (pg/ml), and TNF-α (ng/L) in SHEEP; CRP (mg/L) and fibrinogen (g/L) in PROCARDIS and CRP (mg/L) in IMPROVE] in the healthy participants of the SHEEP, PROCARDIS and IMPROVE studies. Original data were transformed on a natural log (CRP, fibrinogen, IL6, IL8, TNF-α) or square root scale (sIL6R) to achieve and/or improve the normality of the distribution. Individuals with missing data (genotype, biomarker measurement and covariates) were excluded from the analysis. The final number of individuals included in the analysis for each biomarker is reported in the S2 Table. The association of each haplotype with serum/plasma levels of the biomarkers is estimated by a regression parameter and 95%CI where the effect of each haplotype is compared to the most frequent haplotype that THESIAS sets as the intercept in the regression model. The estimated regression parameters were adjusted by age and sex in SHEEP and by age, sex and participating center in the PROCARDIS and IMPROVE studies. Data are reported as estimated change in the circulating levels of the biomarker as compared to the reference haplotype. They represent the antilog/power of the regression parameter and 95%CI and are expressed in the original unit of measurement for each biomarker [20]. The variance in serum/plasma levels of different inflammatory biomarkers explained by *IL6R* haplotypes was estimated from the square of the ratio between the global and the residual standard error, derived from THESIAS´ analysis algorithm.

Risk estimates of MI/CHD associated with *IL6R* haplotypes were expressed as odds ratio (OR) and 95% CI in the SHEEP and PROCARDIS studies and as hazard ratios (HR) and 95% CI in the IMPROVE study. Risk estimates for MI were adjusted for age, sex and residential area in the SHEEP study, and for age sex and participating centers in the PROCARDIS and IMPROVE studies.

Significance threshold was set at p< 0.05 in the SHEEP study and association analyses were replicated in the PROCARDIS and IMPROVE studies. No adjustments for multiple comparisons were made since haplotypes are highly interrelated.

## Results

The demographic characteristics of the three study populations are summarized in Table 1. Overall, prevalence of the cardiovascular risk factors was different among the three populations. Cardiovascular risk factors occurred more frequently among the cases than the controls with the exception of hypertension in the SHEEP study.

**Table 1:** Demographic characteristics of the study participants in the three study populations.

	SHEEP	(N = 2774)	PROCARDIS	(N = 7997)	IMPROVE	(N = 3711)
	Cases	Controls	Cases	Controls	Cases	Referents
	(N = 1231)	(n = 1561)	(N = 5689)	(N = 2308)	(N = 198)	(N = 3318)
**Man/Women**	852/361	1054/507	4262/1425	1746/562	121/77	1571/1747
**Age (mean (95%CI)**	59.2 (58.8–59.6)	59.8 (59.4–60.1)	61.9 (61.7–62.1)	55.1 (54.6–55.4)	65.5 (64.7–66.3)	65.5 (64.7–66.3)
**Hypertension (%)**	46	52	88	43	75	68
**Diabetes (%)**	19	8	19	4	33	26
**Smoking (%)**	49	30	51	21	21	14
**Hyperchol (%)**	43	30	74	18	71	62
**Obesity (%)**	18	12	32	14	25	21

Hyperchol: hypercholesterolemia.

SHEEP: hypertension defined as blood pressure >140/90 mmHg and/or self-reported and/or antihypertensive drug treatment; diabetes defined as glucose levels>6.7mmol/L and/or insulin or hypoglycemic drugs; smoking defined as current smoking in the past two years; hypercholestesterolemia defined as serum level of total cholesterol ≥6.46 mmol/L and/or treatment with lipid lowering drugs; obesity defined as body mass index>30kg/m2.

PROCARDIS: hypertension defined as blood pressure >140/90 mmHg and/or self-reported and/or antihypertensive drug treatment; diabetes defined as self-reported, glucose levels>7.0mmol/L and/or insulin or hypoglycemic drugs; hypercholestesterolemia defined as serum level of total cholesterol ≥6.46 mmol/L and/or treatment with lipid lowering drugs; obesity defined as body mass index>30kg/m2.

IMPROVE: hypertension and hypercholesterolemia were self-reported at the time of enrollment; diabetes defined as self reported and/or glucose levels>7.0mmol/L and/or insulin or hypoglycemic drugs; smoking defined as current smokers; obesity defined as body mass index>30kg/m2

**IL6R haplotypes and serum CRP levels.** Association of *IL6R* haplotypes with serum CRP levels was tested in the control group of SHEEP and replicated in the controls of the PROCARDIS and IMPROVE studies. The relative change in serum CRP levels (mg/L) associated with one copy of each haplotype as compared to the reference haplotype is reported in Table 2. Diplotypes in block 1 were neither associated with serum CRP levels in SHEEP controls, nor in the PROCARDIS study (Table 2).

**Table 2:** Association of *IL6R* haplotypes in block 1 and block 2 with differences in serum CRP levels (compared to the reference haplotype where mean CRP (95%CI) are shown) in controls from the SHEEP and PROCARDIS and in block 2 from the IMPROVE study.

*Block 1*	*SHEEP*	*P*	*PROCARDIS*	*P*			
	*(n = 1088)*		*(n = 2231)*				
**12**	1.15 (1.04–1.25)	reference	1.05 (0.98–1.12)	reference			
**11**	+1.02 (0.96–1.10)	0.46	+1.03 (1.01–1.16)	0.21			
**21**	+1.01 (0.92–1.10)	0.83	+0.98 (0.99–1.11)	0.46			
***Block 2***	***SHEEP***		***PROCARDIS***		***IMPROVE(95%CI)***	***P***	***Haplotype in IMPROVE***
	***(n = 1052)***		***(n = 2231)***		***(n = 3316)***		
**11211**	1.11 (1.02–1.22)	reference	1.05 (0.99–1.13)	reference	0.98 (0.80–1.20)	reference	**−12–1**
**12122**	+1.08 (0.99–1.20)	0.07	+1.03 (0.97–1.09)	0.22	+1.08 (1.02–1.15)	0.004	**−21–2**
**12111**	+1.12 (1.01–1.23)	0.02	+1.06 (1.0–1.12)	0.03	+1.12 (1.05–1.22)	0.0001	**−21–1**
**21111**	+1.07 (0.97–1.16)	0.16	+1.06 (1.04–1.13)	0.03	+1.14 (1.06–1.22)	0.0003	**−11–1**
**12121**	+1.06 (0.89–1.27)	0.46	+1.09 (0.96–1.23)	0.14			

Data represent the mean and relative 95%CI of the difference in serum CRP levels (mg/L) observed in the presence of one copy of each haplotype configurations as compared to the reference haplotype. Genotype data from only three SNPs in block 2 were available in the IMPROVE study (rs7553796 C/A (pairwise LD with rs4553185T/C, r2 = 0.96) rs8192284A/C and rs4072391T/C (pairwise LD with rs7514452T/C r2 = 0.98).

Allele 1 (A) at rs8192284, in haplotype block 2, has been previously associated with higher serum CRP levels as compared to the 2 (C) allele [2–4,6]. As shown in Table 2, in SHEEP, (n = 1052), all haplotypes in block 2 carrying the A allele at rs8192284 were associated with a relative increase of about 1mg/L in CRP serum levels as compared to the reference haplotype 11211(GTCTT), carrying the alternate C allele.

Haplotype 12111 (GCATT) was associated with the largest increase in CRP serum levels [+1.12mg/L (1.01–1.23) p = 0.02] as compared to the reference haplotype, 11211(GTCTT).

In PROCARDIS (n = 2231), haplotype 12111 (GCATT) and haplotype 21111 (ATATT) were associated with an increase in CRP levels, of +1.06 mg/L (1.0–1.12), and +1.06 mg/L (1.0–1.13), respectively, both p = 0.03, as compared to the most common haplotype 11211 (GTCTT).

In IMPROVE (n = 3316), the following three SNPs were used to generate the haplotype in block 2 [rs7553796 C/A (pairwise LD with rs4553185T/C, r2 = 0.96), rs8192284A/C and rs4072391T/C (pairwise LD with rs7514452T/C r2 = 0.98)]. All haplotypes were associated with an average increase of 1 mg/L in CRP levels as compared to reference haplotype, as shown in Table 2.

Overall, *IL6R* haplotypes explained 3% of the variance in serum CRP levels in SHEEP, 4% in PROCARDIS and 3.4% in IMPROVE.

### IL6R haplotypes and plasma fibrinogen levels

*IL6R* haplotypes were tested for association with plasma fibrinogen levels in SHEEP (n = 1319) and PROCARDIS (n = 2230). Haplotypes in block 1 were not associated with fibrinogen levels (S3 Table).

In SHEEP, haplotypes 12122 (GCACC) and 12111 (GCATT) in haplotype block 2 were associated with the largest difference in plasma fibrinogen levels (g/L) as compared to 1.46 (1.41–1.52) at the reference haplotype (GTCTT, 11211) [with an increase (g/L) of 1.06 (+1.02–1.10), p = 0.001 and +1.04 (1.0–1.08), p = 0.02, respectively]. Consistent with this, in PROCARDIS, haplotypes 12122 (GCACC) and 12111(GCATT) were also associated with a relative increase in plasma fibrinogen levels (+1.02 (0.98–1.04) and +1.02 (0.99–1.05) g/L, respectively), as compared to the reference haplotype 11211 (GTCTT) [1.50 (1.43–1.58)], although the difference did not attain statistical significance (p = 0.05).

*IL6R* haplotypes explained 3.5% and 4.4% of the variation in plasma fibrinogen in SHEEP and PROCARDIS, respectively.

### IL6R haplotypes and serum sIL6R, IL6, IL8 and TNF-α levels in SHEEP

No association was found between *IL6R* haplotypes in block 1 and sIL6R (Table 3).

**Table 3:** Association of *IL6R* haplotypes in blocks 1 and 2 with difference in serum sIL6R levels in controls from the SHEEP compared to the reference haplotype where mean sIL6R (95%CI) are shown.

*Block 1* (n = 1037)	sIL6R (ng/ml)	P
**12**	10.4 (9.4–12.7)	reference
**11**	+0.01 (0.006–0.008)	0.26
**21**	−0.002 (0.008–0.03)	0.68
***Block 2* (n = 1004)**		
**11211**	14.7 (12.6–16.8)	reference
**12122**	−1.08 (-0.5–1.7)	<1*10^−6^
**12111**	−0.68 (-0.31–1.18)	<1*10^−6^
**21111**	−0.55 (-0.23–1.0)	<1*10^−6^
**12121**	−0.68 (-0.16–1.55)	0.0001

Data represent the mean and relative 95%CI of the difference in serum sIL6R levels (ng/ml) observed in the presence of one copy of each haplotype configurations as compared to the reference haplotype.

*IL6R* haplotypes in block 2 were associated with differences in serum concentrations of sIL6R (Table 3) in SHEEP (n = 1004). As shown in Table 3, all haplotypes carrying the 1 (A) allele at rs8192284 were associated with reduced serum levels of sIL6R, and *IL6R* haplotypes accounted for 9.2% of the variance of circulating sIL6R levels in SHEEP.

Haplotype 12111 (GCATT) in block 2 was associated with an increase in serum IL8 levels [+1.11 (1–1.2), p = 0.03], as compared to the reference haplotype (S4 Table). No association was observed between haplotypes in block 1 and serum IL8 levels.

Haplotypes in block 1 and in block 2 were not associated with differences in serum levels of IL6 and TNF-α (S4 Table).

**IL6R haplotypes and risk of cardiovascular events.** Table 4 shows the haplotype frequencies in cases and controls and the risk of MI/CHD associated with the *IL6R* haplotypes in the three studies. Haplotype 12111(GCATT) in block 2 was associated with a 7% increase in the MI/CHD risk in the three study populations, although the result did not attain statistical significance. All the other haplotypes in block 1 and block 2 did not show a consistent association with risk cardiovascular events in the three studies.

**Table 4:** *IL6R* haplotype frequencies in cases and controls and risk of MI in SHEEP and of CHD in the PROCARDIS and IMPROVE studies associated with *IL6R* haplotypes.

	*SHEEP*				*PROCARDIS*				*IMPROVE*				
*Block 1*	*Cases*	*Controls*	*OR (95%CI)*	*P*	*Cases*	*Controls*	*OR (95%CI)*	*P*					
	*(n = 1135)*	*(n = 1459)*			*(n = 5689)*	*(n = 2308)*							
**12**	0.39	0.42	1		0.43	0.43	1						
**11**	0.42	0.39	1.12 (0.9–1.3)	0.05	0.37	0.39	0.93 (0.8–1.0)	0.17					
**21**	0.18	0.18	1.04 (0.9–1.2)	0.6	0.19	0.17	0.96 (0.8–1.1)	0.61					
***Block 2***	***Cases***	***Controls***	***OR (95%CI)***	***P***	***Cases***	***Controls***	***OR (95%CI)***	***P***	***Block 2***	***Cases***	***Controls***	***HR (95%CI)***	***P***
	***(n = 1094)***	***(n = 1405)***			***(n = 5689)***	***(n = 2308)***				***(n = 198)***	***(n = 3316)***		
**11211**	0.38	0.38	1		0.38	0.38	1		**−12–1**	0.33	0.35	1	
**12122**	0.19	0.19	0.93 (0.8–1.1)	0.40	0.18	0.19	1.06 (0.8–1.3)	0.35	**−21–2**	0.21	0.22	0.94 (0.7–1.2)	0.68
**12111**	0.18	0.20	1.07 (0.9–1.3)	0.36	0.21	0.20	1.08 (0.9–1.2)	0.20	**−21–1**	0.23	0.21	1.07 (0.8–1.4)	0.61
**21111**	0.16	0.16	0.96 (0.8–1.1)	0.67	0.15	0.16	0.98 (0.9–1.1)	0.79	**−11–1**	0.20	0.19	1.08 (0.8–1.4)	0.48
**12121**	0.03	0.03	0.93 (0.7–1.3)	0.67	0.03	0.03	1.05 (0.8–1.4)	0.35		-	-	-	-

Only diplotypes and haplotypes with a frequency>1% are reported in the table. The most common diplotype in block 1 and the most common haplotype in block 2 are taken as reference categories.

Data from only three tag SNPs were available in the IMPROVE study (rs7553796 C/A (pairwise LD with rs4553185T/C, r2 = 0.96) rs8192284A/C and rs4072391T/C (pairwise LD with rs7514452T/C r2 = 0.98). Haplotype-21–1 represents both 12111 and 12121.

All the other haplotypes in block 1 and block 2 did not show a consistent association with with risk cardiovascular events in the three studies.

## Discussion

The data presented here represent the most comprehensive analysis of the role of *IL6R* haplotypes in the regulation of circulating levels of inflammatory biomarkers and as a risk factor for CHD. They indicate that *IL6R* haplotypes regulate serum levels of CRP, IL8 and sIL6R and plasma levels of fibrinogen in healthy individuals and are associated with a modest, and in this sample size, non-significant association with the risk of CHD in three, large independent European populations.

*IL6R* encodes both the membrane bound receptor and the sIL6R. The membrane bound IL6R regulates the synthesis and release of CRP and fibrinogen from hepatocytes in response to IL6. We observed that haplotypes containing the A allele of rs8192284, which has formerly been associated with higher circulating levels of CRP and fibrinogen as compared to the alternate C allele [2–6,21], are consistently associated with an increase in the circulating levels of CRP and fibrinogen as compared to the reference haplotype.

In the SHEEP study, we were also able to analyze the association of *IL6R* haplotypes with a panel of inflammatory biomarkers. Even if our data are limited to a relatively small number of individuals, they indicate that *sIL6R* haplotypes are not involved in the regulation of TNF-α and IL6, two upstream regulators of IL6R [22,23]. This is consistent with the hypothesis of a sequential regulation of gene expression in the inflammatory cascade, where upstream genes regulate downstream mediators [13]. We did observe a modest effect of one *IL6R* haplotype on IL8 serum levels. IL8 participates in the shedding of the sIL6R from the membrane bound receptor and is also a mediator of IL6 release in the early stage of the inflammatory process [22]. Replication of this association as well as functional studies are warranted to correctly interpret this finding.

In the SHEEP study, *IL6R* haplotypes carrying the A allele at rs8192284 were associated with lower serum levels of sIL6R, as compared to the reference haplotype, and explained about 9% of the variance in serum sIL6R levels. The sIL6R is released in the circulation mainly through the shedding (proteolytic cleavage) of the extracellular domain of the membrane bound IL6R and via the transcription of an alternative spliced mRNA lacking a sequence of 90 base pairs that encodes the transmembrane domain of the membrane bound receptor. The genetic variant rs8192284A/C introduces an Asp to Ala aminoacid change in the membrane bound receptor at the soluble receptor shedding site. It has recently been shown that presence of the CC genotype is associated with an increased shedding of the receptor and an increased expression and release of the alternatively spliced receptor [24]. The contribution of the sIL6R derived from the spliced variant to the total circulating sIL6R is relatively low, however, it has been shown that sIL6R serum levels vary with age and are increased in hepatic disorders and in acute T-cell leukaemia [25]. The assay used in the present study does not discriminate between the two circulating forms of sIL6R, therefore, we cannot analyse the selective association of IL6R haplotypes with each one of the two sIL6R forms. In addition to rs8192284, two SNPs in almost complete LD with two of the SNPs analysed here (rs8192282 in haplotype block 2 and rs12083537 in haplotype block1) have been shown to have a small effect on circulating levels of sIL6R [24]. In our analysis all haplotypes carrying the A allele at rs8192284 in haplotype block 2 contribute equally to reduce receptor serum levels, thus suggesting that rs8192284 and rs8192282 influence serum sIL6R levels independently from each other. Haplotypes in block 1 were not associated with serum sIL6R levels in SHEEP. A recent report indicates that allele A at rs12083537 was associated with a slight increase in serum sIL6R levels and an increased risk of asthma in two large cohorts [26]. These findings indicate that *IL6R* haplotypes may contribute to define genetic risk profiles for inflammatory diseases.

We tested the association of *IL6R* haplotypes with inflammatory biomarkers in the control group of the SHEEP study and used the same strategy in the two replication cohorts, to avoid the confounding effect of the case status.

**Fig 2:**
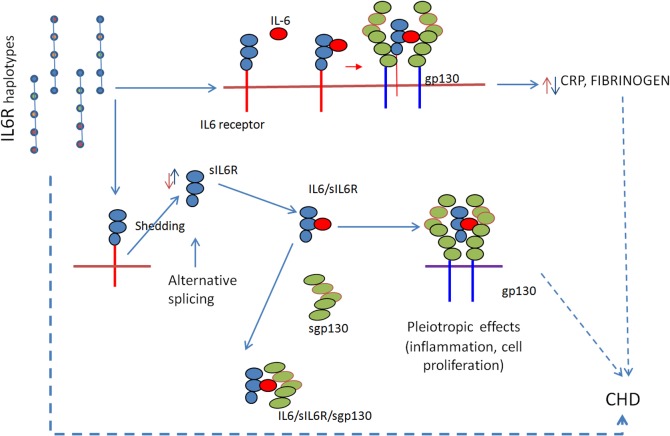
IL6R haplotypes selectively regulate inflammatory biomarkers. **In particular, changes in CRP and fibrinogen are mirrored by inverse changes in sIL6R serum levels.** *Mutations around the shedding site may affect the shedding/alternative splicing and therefore the relative amount of sIL6R in the circulation*. *The sIL6R*, *once released participates in the IL6 transignalling in cells not expressing IL6R (i.e*. *endothelial and smooth muscle cells) and the complex IL6/sIL6R is buffered in the circulation by its natural antagonist*, *sgp130*. *It is possible that the final effect of IL6R haplotypes on the CHD risk depends on the average effect of the association of different SNPs with the CHD risk present in the same haplotype and/or it may reflect changes in the secondary and tertiary structure of the IL6R and sIL6R that may affect the IL6/sIL6R interaction with its downstream mediators*.

Our current data indicate that *IL6R* haplotypes are not significantly associated with risk of CHD in three large European studies. Haplotypes in block 2 contain two SNPs (r2 = 0.46) formerly associated with the risk of CHD. SNP rs4553185T/C (in position 2) in full LD with rs4845625C/T, was associated with a 4% increase in the CHD risk in the CARDIoGRAM-C4D Consortium [1] and SNP rs8192284A/C (in position 3) with a 5% decrease in the risk of CHD [2–4]. The apparent controversy between the single SNP and the haplotype analysis may have several explanations. The biological value of haplotypes differs from that of single SNPs [27,28]. Haplotypes mirror the changes that sequential nucleotide variants impose on the aminoacid sequence and are able to catch subtle changes in the protein function, probably mediated by alteration in secondary or tertiary protein structure, and the effect of a single variant is analyzed in the context of the haplotypic background, regardless of its causality [7,9]. *IL6R* genetic variants have a rather small effect on the MI/CHD risk and populations of larger size may be needed to detect the effect of haplotypes where the effect of alleles with an opposite effect on the CHD risk may average when analyzing the haplotype as a unique allele. At the same time, since shedding of the sIL6R from the membrane bound IL6R receptor is increased in the presence of the rs8192284C allelic variant while it is reduced in the presence of other mutations surrounding the cleavage site [29], we cannot exclude that possibility non-genetic mechanisms related to the sIL6R stability in the circulation are involved in the regulation of biological processes with a central role in atherogenesis. (Fig. 2).

Of note, a relevant clinical implication of this property of haplotypes is the possibility to better identify responders to specific treatments, as shown for example for the beta 2 receptor haplotypes that modify bronchodilator response to beta agonist in asthmatics [27], and to generate individually tailored cardiovascular risk profiles [13].

Several limitations of our study need to be acknowledged. The SNP choice in the present study was based upon tag SNPs in *IL6R* according to the HapMap data release 24 and integrated with SNPs included in the Illumina 200K CardioMetabochip and therefore does not include SNPs recently added to the Hap Map database. Demonstration of association of *IL6R* haplotypes with serum levels of IL6, IL8, sIL6R and TNF-α is limited to the SHEEP study. All the three study populations have been recruited in Europe, and therefore our results cannot be extended to other ethnicities since the pairwise LD values and therefore the haplotype structure differs amongst populations.

In conclusion, our data provide strong evidence that *IL6R* haplotypes selectively regulate circulating levels of inflammatory biomarkers that constitute relevant indicators of cardiovascular risk but have a modest non significant effect on the risk of CHD. They also support the notion that haplotype analysis may identify genetic profiles differentially associated with serum levels of biomarkers and with the risk of complex diseases [30] as well as the responsiveness to pharmacological treatments.

## Supporting Information

S1 Table Haplotypic r2 value of the inferred *IL6R* haplotypes and *IL6R* haplotype frequencies in the SHEEP, PROCARDIS and IMPROVE studies. Diplotype 22 in block 1 has a haplotypic r2 value of 0.60 in both populations and has therefore not been included in the analysis. Data from only three tag SNPs were available in the IMPROVE study (rs7553796 C/A (pairwise LD with rs4553185T/C, r2 = 0.96) rs8192284A/C and rs4072391T/C (pairwise LD with rs7514452T/C r2 = 0.98).
(DOCX)

S2 Table Total number of individuals where the association between *IL6R* haplotypes and circulating biomarkers was analyzed in each of the three studies. Diplotype 22 in block 1 has a r2 haplotypic value of 0.60 in both populations and has therefore not been included in the analysis. Data from only three tag SNPs were available in the IMPROVE study (rs7553796 C/A (pairwise LD with rs4553185T/C, r2 = 0.96) rs8192284A/C and rs4072391T/C (pairwise LD with rs7514452T/C r2 = 0.98).
(DOCX)

S3 Table Association of *IL6R* haplotypes in block 1 with changes in fibrinogen levels in the controls from the SHEEP and PROCARDIS studies. Data represent the mean and 95%CI of the change in fibrinogen plasma levels (g/L) observed in the presence of one copy of each haplotype configurations as compared to the reference haplotype.
(DOCX)

S4 Table Association of IL6R haplotypes in block 1 and block 2 in the controls from the SHEEP study with changes in serum levels of IL6, IL8 and TNF-α. Data represent the mean and relative 95%CI of the change in serum IL6 (ng/ml), IL8 (pg/ml) and TNF-α (ng/L) observed in the presence of one copy of each haplotype as compared to the reference haplotype.
(DOCX)

## References

[1] D Consortium, Deloukas P, Kanoni S, Willenborg C, Farrall M, Assimes TL, et al Large-scale association analysis identifies new risk loci for coronary artery disease. Nature Genetics. 2013;45: 25–33. 10.1038/ng.2480 23202125 PMC3679547

[2] Elliott P, Chambers JC, Zhang W, Clarke R, Hopewell JC, Peden JF, et al Genetic Loci associated with C-reactive protein levels and risk of coronary heart disease. JAMA. 2009;302: 37–48. 10.1001/jama.2009.954 19567438 PMC2803020

[3] Hingorani AD, Casas JP The interleukin-6 receptor as a target for prevention of coronary heart disease: a mendelian randomisation analysis. Lancet. 2012;379: 1214–1224. 10.1016/S0140-6736(12)60110-X 22421340 PMC3316968

[4] Sarwar N, Butterworth AS, Freitag DF, Gregson J, Willeit P, Gorman DN, et al Interleukin-6 receptor pathways in coronary heart disease: a collaborative meta-analysis of 82 studies. Lancet. 2012;379: 1205–1213. 10.1016/S0140-6736(11)61931-4 22421339 PMC3316940

[5] Qi L, Rifai N, Hu FB Interleukin-6 receptor gene, plasma C-reactive protein, and diabetes risk in women. Diabetes. 2009;58: 275–278. 10.2337/db08-0968 18852330 PMC2606885

[6] Rafiq S, Frayling TM, Murray A, Hurst A, Stevens K, Weedon MN, et al A common variant of the interleukin 6 receptor (IL-6r) gene increases IL-6r and IL-6 levels, without other inflammatory effects. Genes Immun. 2007;8: 552–559. 17671508 10.1038/sj.gene.6364414 PMC2668154

[7] Schaid DJ Evaluating associations of haplotypes with traits. Genet Epidemiol. 2004;27: 348–364. 15543638 10.1002/gepi.20037

[8] Gigante B, Bennet A, Leander K, Vikstrom M, De Faire U The Interaction Between Factor 2 Receptor (F2R) and Interleukin 6 Haplotypes and the Risk of Myocardial Infarction in the SHEEP. PloS ONE. 2010;5: e11300 10.1371/journal.pone.0011300 20585578 PMC2891999

[9] Crawford DC, Nickerson DA Definition and clinical importance of haplotypes. Annu Rev Med. 2005;56: 303–320. 15660514 10.1146/annurev.med.56.082103.104540

[10] Farrall M, Green FR, Peden JF, Olsson PG, Clarke R, Hellenius ML, et al Genome-wide mapping of susceptibility to coronary artery disease identifies a novel replicated locus on chromosome 17. PLoS genetics. 2006;2: e72 16710446 10.1371/journal.pgen.0020072 PMC1463045

[11] Baldassarre D, Nyyssonen K, Rauramaa R, de Faire U, Hamsten A, Smit AJ, et al Cross-sectional analysis of baseline data to identify the major determinants of carotid intima-media thickness in a European population: the IMPROVE study. Eur Heart J. 2010;31: 614–622. 10.1093/eurheartj/ehp496 19952003

[12] Reuterwall C, Hallqvist J, Ahlbom A, De Faire U, Diderichsen F, Hogstedt C, et al Higher relative, but lower absolute risks of myocardial infarction in women than in men: analysis of some major risk factors in the SHEEP study. The SHEEP Study Group. J Intern Med. 1999;246: 161–174. 10447785 10.1046/j.1365-2796.1999.00554.x

[13] Gigante B, Vikström M, Strömqvist Meuzelaar L, Chernogubova E, Silveira A, van't Hooft F, et al Variants in the Coagulation Factor 2 Receptor (F2R) Gene Influence the Risk of Myocardial Infarction in Men through an Interaction with IL6 Serum Levels. Thromb Haemost. 2009;101: 943–953. 19404549

[14] Leander K, Gigante B, Silveira A, Vikstrom M, Hamsten A, Hogberg J NAMPT (visfatin) and AKT1 genetic variants associate with myocardial infarction. Clin Chim Acta. 2012;413: 727–732. 10.1016/j.cca.2012.01.002 22251423

[15] Jurinke C, Oeth P, van den Boom D MALDI-TOF mass spectrometry: a versatile tool for high-performance DNA analysis. Mol Biotechnol. 2004;26: 147–164. 14764940 10.1385/MB:26:2:147

[16] Voight BF, Kang HM, Ding J, Palmer CD, Sidore C, Chines PS, et al The metabochip, a custom genotyping array for genetic studies of metabolic, cardiovascular, and anthropometric traits. PLoS genetics. 2012;8: e1002793 10.1371/journal.pgen.1002793 22876189 PMC3410907

[17] Barrett JC, Fry B, Maller J, Daly MJ Haploview: analysis and visualization of LD and haplotype maps. Bioinformatics. 2005;21: 263–265. 15297300 10.1093/bioinformatics/bth457

[18] Gabriel S, Schaffner S, Nguyen H, Moore J, Roy J, Blumenstiel B, et al The structure of haplotype blocks in the human genome. Science. 2002;296: 2225–2229. 12029063 10.1126/science.1069424

[19] Tregouet DA, Garelle V A new JAVA interface implementation of THESIAS: testing haplotype effects in association studies. Bioinformatics. 2007;23: 1038–1039. 17308338 10.1093/bioinformatics/btm058

[20] Bland JM, Altman DG Transformations, means, and confidence intervals. BMJ. 1996;312: 1079 8616417 10.1136/bmj.312.7038.1079 PMC2350916

[21] Danik JS, Pare G, Chasman DI, Zee RY, Kwiatkowski DJ, Parker A, et al Novel loci, including those related to Crohn disease, psoriasis, and inflammation, identified in a genome-wide association study of fibrinogen in 17 686 women: the Women's Genome Health Study. Circ Cardiovasc Genet. 2009;2: 134–141. 10.1161/CIRCGENETICS.108.825273 20031577 PMC2749513

[22] Marin V, Montero-Julian FA, Gres S, Boulay V, Bongrand P, Farnarier C, et al The IL-6-soluble IL-6Ralpha autocrine loop of endothelial activation as an intermediate between acute and chronic inflammation: an experimental model involving thrombin. J Immunol. 2001;167: 3435–3442. 11544336 10.4049/jimmunol.167.6.3435

[23] McKellar GE, McCarey DW, Sattar N, McInnes IB Role for TNF in atherosclerosis? Lessons from autoimmune disease. Nat Rev Cardiol. 2009;6: 410–417. 10.1038/nrcardio.2009.57 19421244

[24] Ferreira RC, Freitag DF, Cutler AJ, Howson JM, Rainbow DB, Smyth DJ, et al Functional IL6R 358Ala Allele Impairs Classical IL-6 Receptor Signaling and Influences Risk of Diverse Inflammatory Diseases. PLoS genetics. 2013;9: e1003444 10.1371/journal.pgen.1003444 23593036 PMC3617094

[25] Horiuchi S, Ampofo W, Koyanagi Y, Yamashita A, Waki M, Matsumoto A, et al High-level production of alternatively spliced soluble interleukin-6 receptor in serum of patients with adult T-cell leukaemia/HTLV-I-associated myelopathy. Immunology. 1998;95: 360–369. 9824498 10.1046/j.1365-2567.1998.00622.x PMC1364401

[26] Revez JA, Bain L, Chapman B, Powell JE, Jansen R, Duffy DL, et al A new regulatory variant in the interleukin-6 receptor gene associates with asthma risk. Genes Immun. 2013;14: 441–446. 10.1038/gene.2013.38 23945879 PMC4233139

[27] Drysdale CM, McGraw DW, Stack CB, Stephens JC, Judson RS, Nandabalan K, et al Complex promoter and coding region beta 2-adrenergic receptor haplotypes alter receptor expression and predict in vivo responsiveness. Proc Natl Acad Sci U S A. 2000;97: 10483–10488. 10984540 10.1073/pnas.97.19.10483 PMC27050

[28] Small K, Mialet-Perez J, Ligget SB Genetic Variation Within the b1-Adrenergic Receptor Gene Results in Haplotype-Specific Expression Phenotypes. J Cardiovasc Pharmacol 2008;51: 106–110. 10.1097/FJC.0b013e31815a958f 18209576

[29] Garbers C, Monhasery N, Aparicio-Siegmund S, Lokau J, Baran P, Nowell MA, et al The interleukin-6 receptor Asp358Ala single nucleotide polymorphism rs2228145 confers increased proteolytic conversion rates by ADAM proteases. Biochim Biophys Acta. 2014;1842: 1485–1494. 10.1016/j.bbadis.2014.05.018 24878322

[30] Pickard BS, Christoforou A, Thomson PA, Fawkes A, Evans KL, Morris SW, et al Interacting haplotypes at the NPAS3 locus alter risk of schizophrenia and bipolar disorder. Mol Psychiatry. 2009;14: 874–884. 10.1038/mp.2008.24 18317462

