# Supplementary material for: Analysis of the Role of Interleukin 6 Receptor Haplotypes in the Regulation of Circulating Levels of Inflammatory Biomarkers and Risk of Coronary Heart Disease
Source: PLoS One. 2015 Mar 17;10(3):e0119980. doi: 10.1371/journal.pone.0119980 (PMC4364007; doi:10.1371/journal.pone.0119980)
Supplement: S1 Table — Diplotype 22 in block 1 has a haplotypic r2 value of 0.60 in both populations and has therefore not been included in the analysis. Data from only three tag SNPs were available in the IMPROVE study (rs7553796 C/A (pairwise LD with rs4553185T/C, r2 = 0.96) rs8192284A/C and rs4072391T/C (pairwise LD with rs7514452T/C r2 = 0.98). (DOCX) [file pone.0119980.s001.docx]

S1 Table. **Haplotypic r2 value of the inferred** *IL6R* **haplotypes and** *IL6R* **haplotype frequencies in the SHEEP, PROCARDIS and IMPROVE studies.**

| *Block 1* | R2 haplotypic value | Haplotype frequency | Haplotype frequency | |  | R2 haplotypic value | Haplotype frequency |
| --- | --- | --- | --- | --- | --- | --- | --- |
|  |  | **SHEEP**  **(n=2594)**  0.41 | | **PROCARDIS**  **(n=7997)** |  |  | **IMPROVE**  **(n=3514)** |
| AT (12) | 0.99 |  |  | 0.43 |  |  |  |
| AA (11) | 0.99 | 0.40 | | 0.37 |  |  |  |
| GA (21) | 0.99 | 0.18 | | 0.19 |  |  |  |
| *Block 2* |  |  | |  | ***Block 2*** |  |  |
| GTCTT (11211) | 0.99 | 0.38 | | 0.38 | **-CC-C (-12-1)** | 0.99 | 0.35 |
| GCACC (12122) | 0.98 | 0.19 | | 0.18 | **-AA-T (-21-2)** | 0.98 | 0.22 |
| GCATT (12111) | 0.98 | 0.19 | | 0.21 | **-AA-C (-21-1)** | 0.98 | 0.22 |
| ATATT (21111) | 0.98 | 0.16 | | 0.16 | **-CA-C (-11-1)** | 0.97 | 0.19 |
| GCACT (12121) | 0.96 | 0.03 | | 0.03 |  |  |  |

Diplotype 22 in block 1 has a haplotypic r2 value of 0.60 in both populations and has therefore not been included in the analysis. Data from only three tag SNPs were available in the IMPROVE study (rs7553796 C/A (pairwise LD with rs4553185T/C, r2=0.96) rs8192284A/C and rs4072391T/C (pairwise LD with rs7514452T/C r2=0.98).
