# Supplementary material for: Analysis of the Role of Interleukin 6 Receptor Haplotypes in the Regulation of Circulating Levels of Inflammatory Biomarkers and Risk of Coronary Heart Disease
Source: PLoS One. 2015 Mar 17;10(3):e0119980. doi: 10.1371/journal.pone.0119980 (PMC4364007; doi:10.1371/journal.pone.0119980)
Supplement: S2 Table — Diplotype 22 in block 1 has a r2 haplotypic value of 0.60 in both populations and has therefore not been included in the analysis. Data from only three tag SNPs were available in the IMPROVE study (rs7553796 C/A (pairwise LD with rs4553185T/C, r2 = 0.96) rs8192284A/C and rs4072391T/C (pairwise LD with rs7514452T/C r2 = 0.98). (DOCX) [file pone.0119980.s002.docx]

S2 Table. Total number of individuals where the association between *IL6R* haplotypes and circulating biomarkers was analyzed in each of the three studies.

|  | | SHEEP (n=1561) | | PROCARDIS (n=2308) | | IMPROVE (n=3318) | |
| --- | --- | --- | --- | --- | --- | --- | --- |
| *CRP* | |  | |  | |  | |
| Missing serum/CRP | | 402 | | 57 | | 2 | |
| CRP=0 | | 1 | | 9 | | 0 | |
| CRP>100 | | 1 | | 0 | | 3 | |
| Missing age | | 0 | | 11 | | 0 | |
| Missing genotype at one/more SNPs in  Haplotype Block1/Block2 | | 89/105 | | 0 | | 2 | |
| Total included in the analysis  Haplotype Block1/Block2 | | 1088/1052 | | 2231/2231 | | 3311 | |
| *Fibrinogen* | |  | |  | | - | |
| Missing plasma/fibrinogen | | 102 | | 58 | | - | |
| Fibrinogen=0 | | - | | 9 | | - | |
| *Missing age* | - | | 11 | | - | |  |
| Missing genotype at one/more SNPs in  Haplotype Block1/Block2 | 98/140 | | 0/0 | | - | |  |
| Total included in the analysis  Haplotype Block1/Block2 | 1361/1319 | | 2230/2230 | | - | |  |
| *sIL6R* |  | |  | |  | |  |
| Missing serum/sIL6R | 457 | | - | | - | |  |
| sIL6R=0 | 2 | | - | | - | |  |
| Missing genotype at one/more SNPs in  Haplotype Block1/Block2 | 65/98 | | - | | - | |  |
| Total included in the analysis  Haplotype Block1/Block2 | 1037/1004 | | - | | - | |  |
| IL6 |  | |  | |  | |  |
| Missing serum/IL6 | 430 | | - | | - | |  |
| IL6=0 | 13 | | - | | - | |  |
| Missing genotype at one/more SNPs in  Haplotype Block1/Block2 | 66/101 | | - | | - | |  |
| Total included in the analysis  Haplotype Block1/Block2 | 1052/1017 | |  | |  | |  |
| IL8 |  | | - | | - | |  |
| Missing serum/IL8 | 438 | |  | |  | |  |
| IL8=0 | 13 | |  | |  | |  |
| Missing genotype at one/more SNPs in  Haplotype Block1/Block2 | 66/100 | |  | |  | |  |
| Total included in the analysis  Haplotype Block1/Block2 | 1054/1020 | |  | |  | |  |
| TNF-α |  | |  | |  | |  |
| Missing serum/TNF-α | 551 | | - | | - | |  |
| TNF-α=0 | 0 | | - | | - | |  |
| Missing genotype at one/more SNPs in  Haplotype Block1/Block2 | 54/86 | |  | |  | |  |
| Total included in the analysis  Haplotype Block1/Block2 | 956/924 | |  | |  | |  |

Diplotype 22 in block 1 has a r2 haplotypic value of 0.60 in both populations and has therefore not been included in the analysis. Data from only three tag SNPs were available in the IMPROVE study (rs7553796 C/A (pairwise LD with rs4553185T/C, r2=0.96) rs8192284A/C and rs4072391T/C (pairwise LD with rs7514452T/C r2=0.98).
