# Supplementary material for: Analysis of the Role of Interleukin 6 Receptor Haplotypes in the Regulation of Circulating Levels of Inflammatory Biomarkers and Risk of Coronary Heart Disease
Source: PLoS One. 2015 Mar 17;10(3):e0119980. doi: 10.1371/journal.pone.0119980 (PMC4364007; doi:10.1371/journal.pone.0119980)
Supplement: S3 Table — Data represent the mean and 95%CI of the change in fibrinogen plasma levels (g/L) observed in the presence of one copy of each haplotype configurations as compared to the reference haplotype. (DOCX) [file pone.0119980.s003.docx]

S3 Table. **Association of *IL6R* haplotypes in block 1 with changes in fibrinogen levels in the controls from the SHEEP and PROCARDIS studies.**

| Fibrinogen (g/L) | SHEEP (n=1361) | p | PROCARDIS (n=2230) | p |
| --- | --- | --- | --- | --- |
| 12 | 1.52  (1.46-1.58) | reference | 1.53  (1.46-1.61) | reference |
| 11 | - 0.98  (0.95-1.01) | 0.23 | -0.98  (0.96-1.01) | 0.28 |
| 21 | -0.99  (0.95-1.02) | 0.65 | -0,97  (0,96-1) | 0.16 |

Data represent the mean and 95%CI of the change in fibrinogen plasma levels (g/L) observed in the presence of one copy of each haplotype configurations as compared to the reference haplotype.
