# Supplementary material for: Analysis of the Role of Interleukin 6 Receptor Haplotypes in the Regulation of Circulating Levels of Inflammatory Biomarkers and Risk of Coronary Heart Disease
Source: PLoS One. 2015 Mar 17;10(3):e0119980. doi: 10.1371/journal.pone.0119980 (PMC4364007; doi:10.1371/journal.pone.0119980)
Supplement: S4 Table — Data represent the mean and relative 95%CI of the change in serum IL6 (ng/ml), IL8 (pg/ml) and TNF-α (ng/L) observed in the presence of one copy of each haplotype as compared to the reference haplotype. (DOCX) [file pone.0119980.s004.docx]

S4 Table. **Association of IL6R haplotypes in block 1 and block 2 in the controls from the SHEEP study with changes in serum levels of IL6, IL8 and TNF-α.**

| *Block 1* | IL6 | P | IL8 | P | TNF-α | P | | |  |  |
| --- | --- | --- | --- | --- | --- | --- | --- | --- | --- | --- |
| 12 | 1.87 (1.66-2.11) | reference | 2.88 (2.55-3.22) | reference | 1.12 (1.06-1.19) | reference | | |  |  |
| 11 | -0.93 (-0.85-1.02) | 0.17 | -1.08 (0.99-1.17) | 0.06 | -0.98 (0.9-1.03) | 0.51 | | |  |  |
| 21 | +1.05 (0.93-1.18) | 0.41 | -1.05 (0.93-1.18) | 0.61 | -0.98 (0.9-1.04) | 0.59 | | |  |  |
| *Block 2* |  |  |  |  |  |  | | |  |  |
| 11211 | 1.91 (1.69-2.15) | reference | 2.88 (2.55-3.25) | reference | 1.09 (1.02-1.17) | reference | | |  |  |
| 12122 | -0.99 (-0.88-1.12) | 0.96 | -0.99 (0.95-1.06) | 0.37 | -0.98 (-0.93-1.03) | | 0.72 | | |  |
| 12111 | -0.93 (-0.82-1.06) | 0.30 | +1.11 (1-1.25) | 0.03 | +1 (0.94-1.07) | | | 0.86 | | |
| 21111 | -0.99 (-0.86-1.13) | 0.88 | +1.05 (0.94-1.18) | 0.35 | + 1.02 (0.95-1.09) | | | 0.48 | | |
| 12121 | -0.86 (-0.71-1.03) | 0.10 | -0.98 (0.80-1.17) | 0.77 | +1.09 (0.97-1.23) | | | 0.14 | | |

Data represent the mean and relative 95%CI of the change in serum IL6 (ng/ml), IL8 (pg/ml) and TNF-α (ng/L) observed in the presence of one copy of each haplotype as compared to the reference haplotype.
